# The mediating role of self-stigma and self-efficacy between intimate partner violence (IPV) victimization and depression among men who have sex with men in China

**DOI:** 10.1186/s12889-019-8125-y

**Published:** 2020-01-03

**Authors:** Liping Peng, Rui She, Jing Gu, Chun Hao, Fengsu Hou, Dannuo Wei, Jinghua Li

**Affiliations:** 1 School of Public Health, Sun Yat-sen University, No.74, Zhongshan second road, Guangzhou, China; 2 Centre for Health Behaviours Research, JC School of Public Health and Primary Care, The Chinese University of Hong Kong, Hong Kong, SAR China; 3 Sun Yat-sen Global Health Institute, Sun Yat-sen University, Guangzhou, China; 4 Department of Public Mental Health, Shenzhen Kangning Hospital, Shenzhen, Guangdong China

**Keywords:** Men who have sex with men, Intimate partner violence, Depression, Self-stigma, Self-efficacy

## Abstract

**Background:**

Previous studies have shown that intimate partner violence (IPV) is prevalent in men who have sex with men (MSM). Mental health problems among MSM with IPV victimization have become a growing concern. The present study examined homosexual self-stigma and self-efficacy as potential mediators of the association between IPV victimization and depression.

**Methods:**

We recruited 578 MSM from 15 cities across China. Participants completed sociodemographic measures, the IPV-GBM (IPV among gay and bisexual men) scale, the Self-Stigma Scale-Short Form (SSS-S), the General Self-Efficacy (GSE) Scale and the Center for Epidemiologic Studies Depression 10 (CES-D-10). We calculated bias-corrected 95% confidence interval (CI) for total, direct and indirect effects using bootstrapping to conduct mediation analyses.

**Results:**

Findings showed that the prevalence of IPV victimization and depression were 32.7% (189/578) and 36.0% (208/578), respectively. Result from mediation analysis using structural equation modeling indicated that the association between level of IPV victimization and depression among MSM was fully mediated by higher homosexual self-stigma and lower self-efficacy. Homosexual self-stigma had a direct effect and an indirect effect via self-efficacy on depression.

**Conclusion:**

The results provided evidence that integrated interventions that reduce self-stigma and foster self-efficacy could be promising approaches to decrease depression among MSM with IPV victimization.

## Introduction

Intimate partner violence (IPV) is generally defined as physical, sexual, psychological or financial harm by a current or former intimate partners or spouse [1, 2], which is a significant public health issue among women. However, IPV is not exclusive to opposite-sex relationship. The emergency of research on men who have sex with men (MSM) has demonstrated that IPV occurs in male-male partnerships at rates similar to or higher than opposite-sex relationship [3]. A recent systematic review concluded that the estimated prevalence of lifetime IPV was 41.2% [95% confidence interval (CI) 32.4–50.1%] among MSM [3]. Studies in China also documented high prevalence of IPV among MSM: ranging from 18.7 to 51.0% for any form of IPV [4–7], 6.6–16.1% for physical IPV, and 5.5–5.7% for sexual IPV. Some studies also show that IPV prevalence is higher in special groups of MSM, such as male sex workers (57.4%) and men who have sex with men and women (37.6%) [4, 7]. IPV experiences can result in a variety of high-risk sexual behaviors and health problems, such as substance use, engagement in unprotected anal intercourse, group sex, transactional sex, HIV infection [6, 7], depression, and suicide behaviors [3]. For instance, a survey among MSM in UK in the PROUD trial from 2012 to 2014 showed that clinically significant depressive symptom prevalence was approximately three times higher in MSM who reported IPV victimization (adjusted prevalence ratios =2.57, 95% CI = 1.71–3.86, *P* < 0.001 for lifetime IPV victimization; adjusted prevalence ratios =2.93, 95% CI = 1.96–4.40, *P* < 0.001 for IPV victimization in last year) [8]. A survey among HIV-negative MSM in Northeastern China in 2014 showed that 18.7% (89/476) of the participants reported being victims of any form of IPV (including physical, psychological and sexual) in the past 3 months and those who had been victims of IPV in the past 3 months were more likely to suffer from symptoms of depression (adjusted odds ratios = 2.8, 95% CI = 1.7–4.5, *P* < 0.05) [6]. Although the issue of IPV and mental health has attracted more attention globally, researches on the relationship between IPV victimization and mental health among MSM in China are still rare. Thus, it is greatly warranted to have a closer look at the association between IPV and depression among MSM population in China.

Although the association between IPV and depression in MSM has been reported in literature, the potential pathways linking IPV and depression have been rarely explored. This is crucial for developing effective interventions to maintain and promote mental health of IPV victims. Lifetime IPV victimization among MSM was strongly associated with self-stigma against sexual minority status, which involves a process of incorporating negative societal views of homosexuality into the self-concept [8, 9]. According to the minority stress theory [10, 11], sexual minority people in a heterosexual society are subjected to chronic stress related to their stigmatization, such as self-stigma and perceived stigma, which are prominent determinants of mental health for minority populations. Empirical evidence also suggested that self-stigma was significantly associated with greater risk of depression among Chinese MSM [12–14], especially among HIV-positive MSM. In addition, several studies have demonstrated that self-stigma was a significant mediator in the association between prejudiced experiences such as victimization and mental distress among sexual minorities [15, 16]. This may suggest a possible mediation effect of self-stigma on the relationship between IPV victimization and depression in MSM [17].

Another potential mediator is self-efficacy, which denotes the belief in one’s capabilities to organize and execute the courses of action required to manage prospective situations [18, 19]. The perception of having personal control and confidence is one important factor in the study of contributors to mental health after exposure to potentially traumatizing events. Extant literature on self-efficacy in IPV victims were mainly focused on financial self-efficacy and condom negotiation self-efficacy among heterosexual women [20, 21]. Few studies have explored the impact of self-efficacy on the mental health of MSM with IPV victimization. The experience of IPV could be particularly harmful for self-worth and self-efficacy, due to the role that the interactions with others play in the development of self-representations [22]. A study conducted among Chinese MSM indicated that a higher level of general self-efficacy was associated with lower levels of depression [23]. General self-efficacy has been found to be a mediator between stressful life events and depressive symptoms among general population [24]. Thus, IPV may deteriorate self-efficacy, which in turn may increase the risk of depression. In addition, previous studies among sexual minority population revealed that self-stigma could weaken one’s self-efficacy [25] and lead to negative health outcomes, such as unhealthy behavior [26] and adverse psychological problems (e.g., depression) [27].

Thus, the present study aimed to investigate the association between IPV victimization and depression among Chinese MSM, and to test the extent to which the association between IPV and depression would be mediated by self-stigma and self-efficacy. We hypothesized that: (1) IPV victimization would be associated with higher self-stigma and lower self-efficacy; (2) self-stigma would be negatively associated with self-efficacy and positively associated with depression; (3) self-efficacy would be negatively associated with risk of depression.

## Method

### Study population and procedure

A cross-sectional survey was conducted from April to June in 2019 in 15 cities across China, including five cities in East China (Sanya, Fuzhou, Hangzhou, Shenzhen and Qingdao), three cities in Midland China (Taiyuan, Changsha and Hefei), four cities in Northeast China (Changchun, Zhengzhou, Harbin and Urumqi), and three cities in West China (Lanzhou, Nanning and Kunming). The selected cities cover all main regions of China and are the main cities in their provinces. Because each city is the first-tier city or provincial capital city of China and their importance are of the same level, so the number of participants recruited by each city is approximately the same (40–50 per city), rather than sampling by population. Participants were recruited through local gay-friendly health consulting organizations and peer referrals. A professional questionnaire research platform (www.wjx.cn) was selected to release the online questionnaire. Participants were firstly briefed about the study purpose, procedure and benefits. Participants provided the informed consent and completed the online questionnaire by clicking to enter the website provided by fieldworkers. The online questionnaire took an average of 15 min to complete. Inclusion criteria were: 1) male aged 18 and above, 2) self-reported anal intercourse with at least one man in the last 6 months, and 3) has or has ever had an intimate partner. Fieldworkers checked the questionnaire upon completion and the questionnaire research platform would also review the logic errors of the questionnaire according to our preset logic rules, and screen out the invalid questionnaire. Each participant who had completed and submitted a qualified questionnaire was offered CNY 15 (about USD 2.5) as a compensation for their time spent on the survey. Of all the 1233 participants approached, 578 eligible participants with complete information were included in our survey, while 573 of them gave up filling midway or fell into the “trap item” which we designed in some of the scales to screen out invalid questionnaires with logic errors, and 82 of them fail to meet the inclusion criteria as reporting no intimate partner.

Ethics approval was obtained from the ethics committee of Sun Yat-sen University (Approval No. [2018] 049).

### Measurement

#### Background characteristic

Sociodemographic information was collected including age, ethnicity (Han or other ethnicity), marital status (single, married, having girlfriend, having boyfriend, divorced, widowed, or others), education level (primary school or below, junior high school, senior high school, undergraduate, or postgraduate), personal monthly income, employment (full-time, part-time, student, unemployed, or retired), sexual orientation (homosexual, heterosexual, bisexual, or not sure), and history of sexually transmitted diseases (STDs).

#### IPV victimization

Five items with reference to IPV-GBM (IPV among gay and bisexual men) scale were developed to assess the IPV, which corresponded to five different domains: physical, sexual, monitoring, controlling and emotional IPV [28]. Example items to assess physical and sexual IPV are “Have any of your intimate partners ever tried to hurt you? This includes hitting you, punching you, kicking you, slapping you, pushing or shoving you, damaging your property, and other physical threats.” and “Have any of your intimate partners ever force you to do something sexually that you didn’t want to do? This includes forcing oral or anal intercourse, forcing you to have sex with someone else, refusing to wear a condom during sex when you requested to use, or any other sexual behavior that makes you feel uncomfortable.” Response to each item is categorized as Yes or No (Yes is coded as 1, and No is coded as 0). The variable of “level of IPV victimization” was a continuous variable, which added up the score of all five items, with a total mean score of 0.62 (standard deviations, SD = 1.11, range = 0–5) in this sample. The variable of “any IPV victimization” was defined as the presence of any of the investigated five types of IPV and was a dichotomous variable, which was coded as “1” for the participants who responded “Yes” to any one of the five items, and “0” for the participants who responded “No” to all five items. “Any IPV victimization” was used only in the descriptive analysis to calculate the prevalence of IPV victimization. In the correlation analysis and SEM analysis, we used “level of IPV victimization”.

#### Homosexual self-stigma

The 9-item Self-Stigma Scale-Short Form (SSS-S) was used to measure the internalized stigma that was attributed to being homosexual self-stigma, which has been validated among Chinese sexual minority populations [29]. It consisted of three subscales: Cognitive (3 items), affective (3 items) and behavioral (3 items). An example item for cognitive subscale was “My identity as a gay is a burden to me.” An example item for affective subscale was “I fear that others would know that I am a gay.” An example item for behavioral subscale was “I avoid interacting with others because I am a gay.” Reponses were rated using a 4-point Likert scale ranging from 1 (strongly disagree) to 4 (strongly agree). Average scores of all items were used, with a higher score indicating a stronger sense of self-stigma. The mean score of SSS-S in this sample was 1.94 (SD = 0.59, range = 1–4). The Cronbach’s alpha was 0.91 in the present sample.

#### Self-efficacy

The General Self-Efficacy (GSE) Scale was used to assess self-efficacy. The GSE scale includes 10 items and has been adapted to Chinese MSM [30, 31]. Sample items include “Thanks to my resourcefulness, I can handle unforeseen situations.” and “I can always manage to solve difficult problems if I try hard enough.” Possible responses were 1 = not at all true, 2 = hardly true, 3 = moderately true, and 4 = exactly true. Items were then summed to create a composite score for self-efficacy ranging from 10 to 40, with higher score indicating higher level of self-efficacy. The Cronbach’s alpha was 0.93 in the present sample.

#### Depression

The Center for Epidemiologic Studies Depression 10 (CES-D-10) was used to measure depressive symptomatology by asking participants about their feelings of sadness and loneliness, difficulties in sleeping and concentrating, lost of interest and hope in the past week. The scale has been validated among Chinese MSM [32–34]. Symptom frequency was reported on a 4-point Likert-type scale ranging from never (0) to nearly every day (3). Each item score was summed up to generate the total score ranging from 0 to 30 and depression was taken as a continuous variable in the correlation analysis and structural equation modeling (SEM). The higher the score, the more severe the depression. But a cut-off of 10 was used to indicate the presence of probable depression [35]. The Cronbach’s alpha was 0.89 in the present sample.

### Statistical analysis

Data were exported directly from the online questionnaire system and analyzed using SPSS 25.0 (SPSS Inc., Chicago, IL). First, chi-square tests were used to investigate differences in prevalence of depression and IPV among participants with different demographic characteristics. Second, spearman’s correlations were conducted to test the relationships between IPV, self-stigma, self-efficacy and depression. Third, structural equation modeling (SEM) was performed by AMOS 22.0 using maximum likelihood method to test the hypothesized mediation model. SEM requires a priori specification of both measurement and structural models. Confirmatory factor analysis (CFA) was firstly conducted to assess the goodness of fit of the measurement model for latent variables including homosexual self-stigma, self-efficacy, and depression. In the next step, the proposed SEM model was examined to test the mediation effects of self-stigma and self-efficacy in the association between IPV and depression. Background variables that were significantly associated with depression were controlled as covariates. To evaluate the overall model fit, we used indices including χ2/df ratio, comparative fit index (CFI), incremental fit index (IFI), and root-mean-square error of approximation (RMSEA). For each index, the following criteria were applied: (1) χ2/df ratio values less than 3 indicates a good model fit [36]; (2) CFI and IFI values greater than 0.9 indicates a good model fit [37]; and (3) for RMSEA, a value between 0.05 and 0.08 indicates an acceptable model fit [38]. Modification indices were inspected to discover the source of the lack of fit, and the model was adjusted accordingly. The total, direct, and indirect effects of the mediation model were estimated using bootstrapping, which has higher power than the commonly used Sobel test or causal steps approach [39]. Bias-corrected 95% CI for each direct and indirect path were reported based on 5000 bootstrap samples. The level of statistically significance was set *p* value< 0.05 (two sided).

## Result

### Background characteristics

Among the 578 participants, 40.5% (*n* = 234) were younger than 25 years old; the mean age was 28.6 (±7.2). Of those sampled, 90.8% (*n* = 528) were Han ethnicity; 54.3% (*n* = 314) had attended university or above; 8.1% (*n* = 47) were married with a woman; 15.7% (*n* = 91) had had a monthly personal income of less than 1000 CNY (160 USD); 66.1% (*n* = 382) had full-time jobs; 81.3% (*n* = 470) had reported their sexual orientation as homosexual; 22.5% (*n* = 130) reported a history of STDs (Table 1).

**Table 1:** Demographic information of participants

Demographic characteristic	*N*=578	IPV Victimization							*p*^a^	Depression		*p*^a^
		Physical *n*=55 (9.5%)	Sexual *n*=67 (11.6%)	Monitoring *n*=87 (15.1%)	Controlling *n*=53 (9.2%)	Emotional *n*=99 (17.1%)	Any *n*=189 (32.7%)	Prevalence (row%)		Yes (*n*=208)	Prevalence (row%)	
Age (years old)												
18–25	234	18	24	28	23	44	84	35.9	0.140	106	45.3	<0.001
26–45	329	36	43	57	29	54	103	31.3		99	30.1	
>45	15	1	0	2	1	1	2	13.3		3	20.0	
Ethnicity												
Han	528	50	59	80	51	86	169	32.0	0.250	184	34.8	0.064
Others	50	5	8	7	2	13	20	40.0		24	48.0	
Education level												
Below university	264	26	28	43	26	45	87	33.0	0.904	97	36.7	0.728
University or above	314	29	39	44	27	54	102	32.5		111	35.4	
Marital status												
Married	47	8	8	10	4	12	14	29.8	0.657	19	40.4	0.508
Unmarried	531	47	59	77	49	87	175	33.0		189	35.6	
Personal monthly income (CNY)												
≤1000	91	8	14	7	5	9	24	26.4	0.233	37	40.7	0.008
1001–3000	148	10	14	18	17	25	51	34.5		66	44.6	
3001–6000	238	29	31	48	22	54	86	36.1		80	33.6	
>6000	101	8	8	14	9	11	28	27.7		25	24.8	
Employment												
Full-time	382	36	41	62	39	65	122	31.9	0.802	129	33.8	0.199
Part-time	45	4	6	10	4	11	17	37.8		14	31.1	
Student	119	11	16	11	4	18	38	31.9		52	43.7	
Unemployed/ Retired	32	4	4	4	6	5	12	37.5		13	40.6	
Sexual orientation												
Homosexual	470	40	54	73	44	75	154	32.8	0.508	172	36.6	0.812
Heterosexual	2	0	0	0	0	0	0	0.0		1	50.0	
Bisexual	91	13	12	14	9	22	32	35.2		31	34.1	
Not sure	15	2	1	0	0	2	3	20.0		4	26.7	
History of STDs												
No	448	40	50	69	43	74	149	33.3	0.594	152	33.9	0.056
Yes	130	15	17	18	10	25	40	30.8		56	43.1	

^a^Chi-square tests

### IPV prevalence

32.7% of the MSM participants reported at least one form of IPV victimization (*n* = 189). Emotional IPV was most frequently reported type (17.1%), followed by ‘monitoring’ (15.1%), ‘sexual’ (11.6%), ‘physical’ (9.5%), and ‘controlling’ (9.2%). There was no statistically significant difference in terms of any IPV victimization prevalence rate between each group (Table 1).

### Depression prevalence

Prevalence of probable depression was 36.0% (208/578) in the present study. It varied with age and personal monthly income in this sample. Depression prevalence was the highest among those aged 18–25 years old (45.3%), followed by 26–45 years old (30.1%) and lowest among those aged above 45 years old (20.0%). Depression prevalence was the highest among those had personal monthly income of 1001–3000 CNY (44.6%), followed by ≤1000 CNY (40.7%), 3001–6000 CNY (33.6%) and > 6000 CNY (24.8%) (Table 1).

### Correlations between variables

Means, standard deviations, and correlations between IPV, homosexual self-stigma, self-efficacy, and depression are presented in Table 2. The mean score for the three subscales of self-stigma was 2.13 (SD = 0.70), 1.97 (SD = 0.65), and 1.72 (SD = 0.58), for affective, cognitive, and behavioral respectively. The mean score of general self-efficacy scale and depression scale (CES-D-10) was 27.45 (SD = 6.00) and 7.55 (SD = 5.75), respectively. We found that IPV victimization was significantly positively correlated with depression (r = 0.206, *p* < 0.01). All the hypothesized mediating variables were significantly correlated with independent variables (IPV victimization) and dependent variables (depression) (Table 2).

**Table 2:** Mean, standard deviation of the variable and correlation between variables in the study

Variables	IPV Victimization	Self-efficacy	Depression	Self-stigma	Mean	SD	Range
IPV Victimization	–				0.62	1.11	0–5
Self-efficacy	−.217^**^	–			27.45	6.00	10–40
Depression	.206^**^	−.536^**^	–		7.55	5.75	0–30
Self-stigma	.118^**^	−.363^**^	.317^**^	–	1.94	0.59	1–4

^**^ *p* < 0.01

### Mediation analysis by SEM

#### Measurement model

As illustrated in Table 3, all of the standardized factor loading of the measurement model were all statistically significant at the level of *p* < 0.001, which ranged from 0.743 to 0.948. The test of the measurement model resulted in the following statistical values: χ2/df ratio = 4.29, CFI = 0.96, IFI = 0.96, RMSEA = 0.075 (90% CI = 0.064, 0.087). With inspection of modification indices (MI), correlation path between self-efficacy parcels residuals (between “parcel 1e” and “parcel 3e”) and depression parcels residuals (between “parcel 2d” and “parcel 3d”) with the largest MI values were added. The modified measurement model yielded a satisfactory model fit: χ2/df ratio = 2.91, CFI = 0.98, IFI = 0.98, RMSEA = 0.058 (90% CI = 0.046, 0.070).

**Table 3:** Unstandardized and standardized loading for measurement model

Parameter estimate	Unstandardized Estimate	Standardized Estimate
stigma → Affective	1	0.902
stigma → Cognitive	0.975***	0.948
stigma → Behavioral	0.698***	0.767
efficacy → Parcel 1e^a^	1	0.866
efficacy → Parcel 2e	1.103***	0.905
efficacy → Parcel 3e	1.487***	0.859
depression → Parcel 1d	1	0.796
depression → Parcel 2d	1.254***	0.820
depression → Parcel 3d	1.008***	0.743

^a^ Items of General Self-Efficacy scale and Center for Epidemiologic Studies Depression 10 were randomly divided into three parcels

*** *p* < 0.001

#### Structural model

**Fig. 1:**
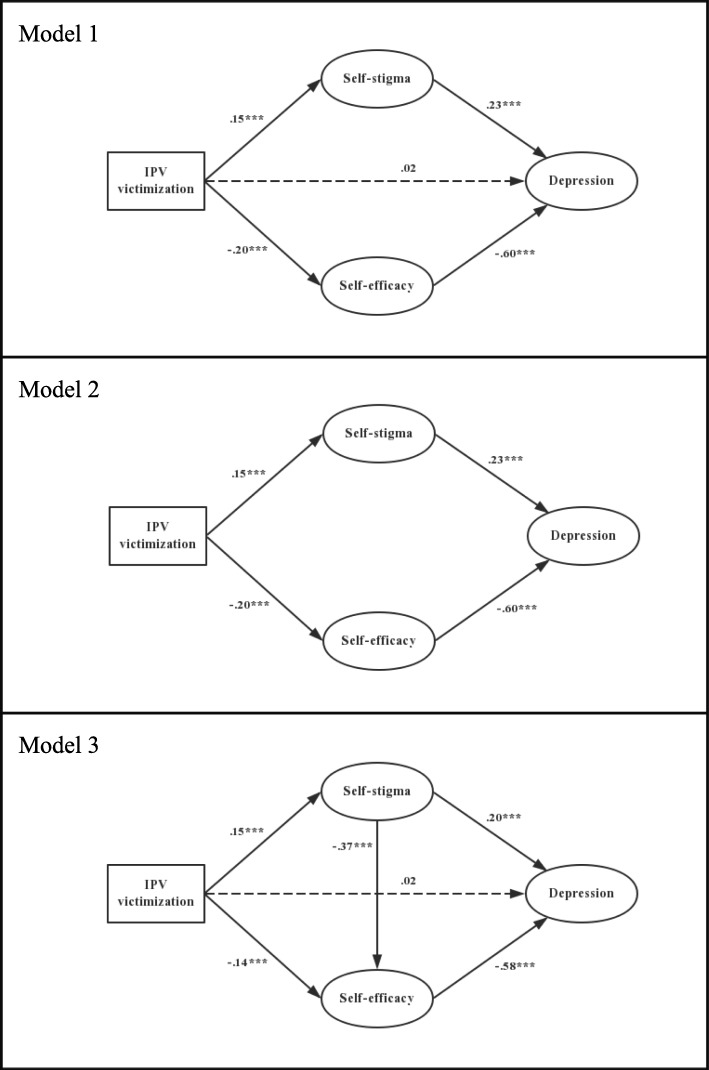
Hypothesized structural models (Model 1, 2 and 3). Note: All path coefficients shown were standardized. *** *p* < 0.001. Age and income have been controlled for in all SEM analyses and were not drawn in the figure

Three models representing different versions of potential mediation roles of self-stigma and self-efficacy between IPV and depression are depicted in Fig. 1. Model 1 was a parallel mediation model between IPV and depression, including the direct path and indirect paths via self-stigma and self-efficacy. Model 2 (to Model 1) was a fully indirect set of paths from IPV to depression via self-stigma and self-efficacy. This model excluded the direct effect to see if it fits the data better. Model 3 (to Model 1) added a path from self-stigma to self-efficacy. This was designed to check whether some of the association between self-stigma and depression were indirect.

Results of these three model tests are presented in Table 4. Model 1 explained 43.7% of the variance in depression but the model fit was unsatisfactory [χ2/df ratio = 4.31, CFI = 0.96, IFI = 0.96, RMSEA = 0.076 (90% CI 0.065, 0.087)]. The direct path from IPV victimization to depression was not statistically significant.

**Table 4:** Goodness-of-fit of hypothesized structural model

Models	CFI	IFI	RMSEA	χ2/df	χ2	df
Model 1	0.96	0.96	0.076	4.31	198.43	46
Model 2	0.96	0.96	0.075	4.23	198.83	47
Model 3	0.98	0.98	0.055	2.75	123.80	45
		Changes in χ2		Changes in df		*p* value
Comparisons						
Model 2 versus model 1		0.40		1		0.527
Model 3 versus model 1		74.63		1		< 0.001

Model 2 accounted for 43.5% of the variance in depression and the model fit did not have significant improvement by excluding the direct path from IPV to depression (*p* = 0.527, Table 4). The association between IPV victimization and depression was fully mediated by homosexual self-stigma and self-efficacy. Model 2 yielded an unsatisfactory fit [χ2/df ratio = 4.23, CFI = 0.96, IFI = 0.96, RMSEA = 0.075 (90% CI 0.064, 0.086)]. The standardized path coefficients of Model 2 are also presented in Fig. 1.

The fit of Model 3 improved significantly as compared to Model 1 (*p* < 0.001, Table 4). Homosexual self-stigma was significantly associated with self-efficacy. Model 3 yielded a satisfactory fit [χ2/df ratio = 2.75, CFI = 0.98, IFI = 0.98, RMSEA = 0.055 (90% CI 0.044, 0.067)]; the overall Model 3 explained 47.8% of the variance in depression. All standardized path coefficients of Model 3 were statistically significant (range: 0.14 to − 0.58, all *p* < 0.001) except for the direct path from IPV to depression.

Model 3 was thus selected as the final model. Bootstrapping was used to estimate the indirect effect of self-stigma and self-efficacy. Three significant indirect paths from IPV and depression were found, 1) through self-stigma, the indirect effect was 0.031 (95% CI: 0.008, 0.069); 2) through self-efficacy, the indirect effect was 0.084 (95% CI: 0.032, 0.145); and 3) through self-stigma and self-efficacy, the indirect effect was 0.033 (95% CI: 0.020, 0.034) (Table 5).

**Table 5:** Summary of total, direct, and indirect effects of the mediation model

Mediation model	Effect (95% CI)	*p* value
Direct effect		
IPV → depression	0.024 (−0.049,0.103)	0.512
Indirect effect	0.148 (0.096, 0.199)	< 0.001
IPV victimization → self-stigma → depression	0.031 (0.008, 0.069)	< 0.001
IPV victimization → self-efficacy → depression	0.084 (0.032, 0.145)	< 0.001
IPV victimization → self-stigma → self-efficacy → depression	0.033 (0.020, 0.034)	< 0.001
Total effect	0.172 (0.081, 0.266)	< 0.001

## Discussion

The study examined the association between IPV victimization and depressive symptoms among Chinese MSM and tested the mediation roles of homosexual self-stigma and self-efficacy. The findings showed that prevalences of lifetime IPV victimization and probable depression were high among Chinese MSM. The mediation model further revealed the association between level of IPV victimization and depression was fully mediated via higher homosexual self-stigma and lower self-efficacy.

The prevalence of IPV victimization was 32.7% (189/578), which was higher than the prevalence reported by two prior surveys among MSM in China (29.8 and 24.3%) [5, 7]. Different from previous studies, the present study included only eligible participants who had or had ever intimate partners, and used a more comprehensive assessment of IPV that involved five different domains. Thus the result could be partially attributed to the cross-study variations in methodology (e.g., sample characteristics, definition and measurement of IPV). We also found a prevalence of 36.0% of depression in the MSM sample, which is comparable to the prevalence reported in previous studies among MSM in China [40, 41]. Younger age and lower income level were associated with increased risk of depression, corroborating with prior studies among Chinese MSM [42, 43]. MSM commonly suffer from stress or rejection due to their sexual identity from family [44]. A qualitative study also showed that internalized homophobia was higher when the MSM population were younger and concealment strategies are often used by young MSM instead of coming out to avoid discrimination and violence associated with stigmatized identity [45]. A study showed that Younger MSM had a higher prevalence of anxiety than older MSM [41], which was also strongly linked to depression. In addition, young MSM were more likely to have sexual risk behaviors and have lower utilization of mental health care, which may further aggravate their psychological problem [46]. Personal income is also associated with depression, which might be partially explained by the financial hardship, discrimination and social stress related to poverty [47]. Mental health interventions and services therefore should be prioritized for younger and poorer MSM, who might be more vulnerable and lack of resources to cope with mental distress.

Consistent with previous studies [48, 49], higher level of IPV victimization was associated with increased risk of depression. The findings further revealed that self-stigma and self-efficacy fully mediated the association between level of IPV and depression. This indicated that IPV experiences increased internalized stigma towards self and was detrimental to self-efficacy, which in turn greatly increased the risk of depression, and psychological interventions to manage negative emotions about one’s sexual minority identity and disrupt negative cognitive appraisals may be helpful to prevent depression in MSM who were IPV victims. Our results provided empirical support for the minority stress model, which posits that self-stigma might be induced by external negative experiences and is an important factor in the development of psychopathology among sexual minorities.

In addition, higher level of IPV and self-stigma was associated with lower self-efficacy and for the first time, self-efficacy was confirmed as a mediator in the association between IPV and depression among MSM population. It seemed that, the violent treatment by intimate partners and negative attitudes towards self could be particularly harmful to the personal sense of competence or confidence in managing problems. These findings corroborate a previous study that supported a similar serial mediation model of self-stigma and self-efficacy in the association between prejudiced events and physical health among sexual minority populations [50]. In fact, several studies have suggested that general self-efficacy may be a source of resilience for people involved in aggressive intimate relationships [51]. Thus the results underscored the importance to increase self-efficacy, for example via personal empowerment and cognitive-behavior therapy, in order to reduce the negative impacts of IPV and stigma on mental health in counseling interventions and psycho-educational programming targeted at MSM.

The study has several limitations. First, given the cross-sectional nature of the present study, we cannot infer any conclusive causal relationships which demands longitudinal studies to further reveal the causalities between these variables. Second, the results may not be generalized to other cultures and populations. Third, reporting bias may exist due to the nature of self-reported data. Fourth, although we recruited participants from 15 cities, selection bias may exist as participants were recruited from local gay-friendly organizations using convenience sampling. Local community organizations could be an importance source of social support and thus participants who had close ties to the organization may have better mental health than those who did not. In addition, majority of the study participants were urban residents and may not be representative of general MSM population. Fifth, IPV-GBM used in the present study has not yet been validated in Chinese MSM and further research is warranted to explore the application and psychometric properties in Chinese population.

## Conclusion

In sum, this study is among one of the first studies to examine the mediation role of homosexual self-stigma and self-efficacy between IPV victimization and depression among MSM in China. Both IPV victimization and depression prevalence rates were high among Chinese MSM, which warranted tailored health interventions and services for MSM. The association between IPV victimization and depression was fully mediated by higher homosexual self-stigma and lower self-efficacy. Homosexual self-stigma had a direct effect and an indirect effect via self-efficacy on depression. The results provided implications that integrated interventions that eliminate self-stigma and foster self-efficacy could be promising approaches to reduce depression among MSM with IPV victimization.

## Data Availability

The datasets used in the study are available from the corresponding author on reasonable request.

## Abbreviations

CES-D-10: The Center for Epidemiologic Studies Depression 10
CFA: Confirmatory factor analysis
CFI: Comparative fit index
CI: Confidence interval
GSE: General Self-Efficacy
IFI: Incremental fit index
IPV: Intimate partner violence
IPV-GBM: Intimate partner violence among gay and bisexual men scale
MSM: Men who have sex with men
RMSEA: Root-mean-square error of approximation
SEM: Structural equation modeling
SSS-S: The Self-Stigma Scale-Short Form
STDs: Sexually transmitted diseases
